# Sol–Gel Synthesis of SnO_2_/Zn_2_–SnO_4_ Heterostructure for the Photocatalytic Degradation of Malachite Green

**DOI:** 10.1021/acsomega.5c13476

**Published:** 2026-02-26

**Authors:** Paulino V. M. Muguirrima, Romuald T. Doumbi, Mario A. M. Castro, Camila Louyse Oliveira da Rocha, Armando Monte Mendes, Theresa B. O. Nunes, Mauricio R. D. Bomio, Fabiana V. Motta, Marcio A. Correa, Carlos Alberto Paskocimas, Antonio Eduardo Martinelli

**Affiliations:** † Postgraduate Program in Materials Science and Engineering, 28123 Federal University of Rio Grande do Norte, Natal, RN 59078-970, Brazil; ‡ LSMC, Metal Ceramic Systems Laboratory, Department of Materials Science and Engineering, Federal University of Rio Grande do Norte, UFRN, P.O. Box 1524, Natal, RN 59078-970, Brazil; § Department of Physics, Federal University of Rio Grande do Norte, Natal, RN 59078-970, Brazil; ∥ LSQM, Laboratory of Chemical Synthesis of Materials, Department of Materials Science and Engineering, Federal University of Rio Grande do Norte, UFRN, P.O. Box 1524, Natal, RN 59078-970, Brazil; ⊥ Faculty of Science and Technology, Zambezi University, Beira 2100, Mozambique

## Abstract

The discharge of contaminated effluents containing organic dyes is a persistent environmental issue due to their toxicity, stability, and resistance to biodegradation. Among these dyes, malachite green (MG) is particularly concerning because of its widespread industrial use and severe ecological and health impacts. To address this problem, SnO_2_/Zn_2_–SnO_4_ heterostructures were synthesized via sol–gel and evaluated for MG degradation under ultraviolet irradiation. The SnO_2_/Zn_2_–SnO_4_ catalyst has been characterized by X-ray diffraction (XRD), photoluminescence (PL), field-emission scanning electron microscopy coupled with energy-dispersive spectroscopy (FE-SEM-EDS), ultraviolet–visible (UV–vis) spectroscopy, Brunauer–Emmett–Teller method, and electrochemical impedance spectroscopy (EIS). Structural properties revealed that the catalyst presents a polycrystalline structure and crystallite sizes in the range of 11–25 nm. The band gap energy of the Sn:Zn (1:2) catalyst was 2.88 eV, which was lower than that of pure SnO_2_ (3.33 eV), indicating enhanced light absorption. MG photocatalysis degradation tests were conducted under ultraviolet irradiation. The ZnSn 1:2 sample achieved a degradation efficiency of approximately 96% after 100 min, while the pure SnO_2_, ZnSn 1:4, and ZnSn 1:6 samples reached only 81%, 88%, and 92%, respectively. This could be due to the presence of h^+^ and ^•^OH species, which were identified as the most active radicals during the photocatalytic process. Furthermore, the 1:2 ZnSn photocatalyst demonstrated good stability and maintained its photocatalytic performance after five successive degradation cycles. These results indicate that the Zn/Sn 1:2 ratio results in the highest photocatalytic efficiency, confirming the superior effect of Zn_2_–SnO_4_ structure in enhancing charge separation and accelerating the degradation of malachite green compared with the other compositions.

## Graphical abstract

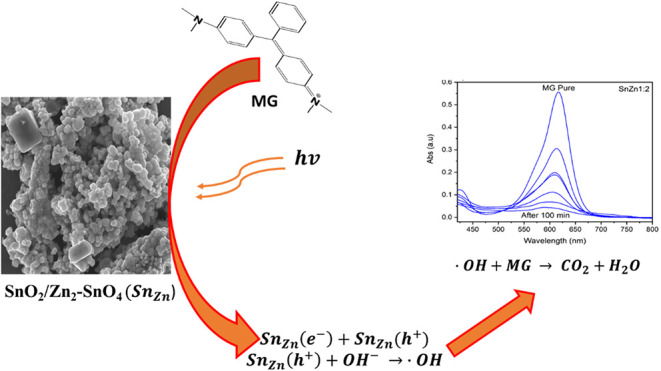

## 1 Introduction

Currently, there is a great need to preserve the planet’s natural resources, especially water resources, which are increasingly polluted by the direct discharge of industrial effluents.^1,2^ One of the sectors that contributes most to the pollution of natural waters is the textile industry, particularly during fabric dyeing, which involves a large amount of water and the use of dyes with toxic and carcinogenic characteristics.^2^ At the end of the process, dye molecules that have not bonded to the fabric fibers remain in solution and are often discharged directly into water bodies. It is estimated that 10–15% of the total dye production is lost, causing not only intense water coloration but also health issues for local organisms and humans.^2,3^ Malachite green (MG) is a cationic dye widely used as a colorant in the textile industry and as a biocide extensively applied in aquaculture systems for the treatment of fungal and parasitic infections worldwide.^3^ However, the toxic and potentially carcinogenic effects of this compound have raised serious concerns regarding its safety and continued application. Prolonged exposure to MG through direct contact, elevated temperature, or higher concentrations makes the compound progressively more hazardous.^4^

Since dyes are resistant to conventional industrial effluent treatment methods, such as biological processes, membrane filtration, coagulation, adsorption, and ion exchange, alternative approaches have been investigated.^5–7^ Among the most recent techniques, the so-called advanced oxidation processes (AOPs) have proven to be promising alternatives for treating these effluents. AOPs are based on the in situ production of hydroxyl radicals (^•^OH), which, due to their high standard redox potential (2.73 V vs NHE) and strong oxidative capacity, are capable of degrading a wide range of organic molecules.^8,9^ AOPs are generally classified into homogeneous and heterogeneous systems. In homogeneous AOPs, the oxidizing species are generated within a single liquid phase—typically through the use of reagents such as hydrogen peroxide or ozone combined with catalysts like Fe^2+^ (as in the Fenton reaction). In contrast, heterogeneous AOPs employ a solid catalyst, often a semiconductor material that activates in the presence of ultraviolet or visible light to produce hydroxyl radicals on its surface. Processes such as Fenton-like reactions, ozonation, and heterogeneous photocatalysis fall under this category.^10,11^ Heterogeneous photocatalysis, in particular, relies on the excitation of electrons from the valence band to the conduction band of the semiconductor upon light absorption. This band gap energy determines the wavelength range required for activation and directly influences the photocatalyst’s efficiency. Consequently, semiconductors with narrower band gaps can utilize visible light more effectively, enhancing photocatalytic activity and energy efficiency.^12^ The main advantage of this method is the potential for mineralizing organic compounds, breaking them down into simple products such as carbon dioxide, water, and inorganic ions.^12^ Among the group of semiconducting oxides, tin oxide (SnO_2_) stands out as an n-type metallic oxide semiconductor with a band gap of approximately 3.6 eV,^13^ originating mainly from cassiterite. Although stoichiometric SnO_2_ exhibits nearly insulating behavior, it is precisely its electrical characteristics, combined with its optical properties, that attract interest in this semiconductor. SnO_2_ is an outstanding material due to its excellent chemical and high photocatalytic activity.^13^ Special attention is paid to the photocatalytic effect of modified SnO_2_. Nevertheless, the main limitation of the SnO_2_-based bulk materials used as photocatalysts is a large band gap (3.6 eV at 300 K), which restricts its photoresponse to the ultraviolet region. To overcome this constraint, the tin oxide band gap can be tuned by introducing dopant elements such as antimony, indium, fluorine, and zinc, which can significantly improve the chemical and physical properties of SnO_2_.^14,15^ Besides increasing the number of available charge carriers, doping semiconductor nanostructures with metal oxides enhances hole-transfer kinetics and accelerates the photocatalytic efficiency in pollutant degradation.^15^

The heterostructure made by Zn and SnO_2_ is a wide class of materials that includes particulate substances, nanoparticles, which have one dimension less than 100 nm at least.^16,17^ Zn and SnO_2_-based materials have been utilized as antibacterial agents, photovoltaic panels, catalysts, solar cells, transparent electrodes, and gas sensors.^18^ Among various modified SnO_2_ materials, Zn has shown remarkable potential for photocatalytic applications. The incorporation of Zn^2+^ ions into the SnO_2_ lattice improves its catalytic ability through several mechanisms: (i) doping with metal ions enhances the catalytic activity of SnO_2_ by modifying its electronic structure; (ii) it reduces the band gap energy, allowing greater utilization of visible light; (iii) the substitution of Sn^4+^ (*r* = 0.69 Å) by Zn^2+^ (*r* = 0.74 Å) introduces defect sites and oxygen vacancies, which serve as active centers for charge separation; and (iv) Zn^2+^ acts as an acceptor level near the valence band, facilitating hole accumulation and improving charge transfer to adsorbed species. These effects collectively enhance the photocatalytic efficiency of Zn-modified SnO_2_ for environmental and energy-related applications.^15^

Different methods have been used to prepare SnO_2_ thin films, such as chemical vapor deposition,^19^ spin coating,^20^ radio frequency magnetron sputtering,^21^ chemical bath deposition,^9^ spray pyrolysis, sol–gel,^22^ and thermal evaporation, among others.^22^ However, the sol–gel technique remains an appropriate and economical processing approach.^23^ Recently, some works reported the removal of MG from water by photocatalysis. Jabenn et al.^24,25^ demonstrated that nanostructured LaFeO_3_ achieved a removal efficiency of 80% of MG under visible light irradiation. Serbout et al.^26^ also demonstrated that a SnO_2_/TiO_2_ heterostructure improves the degradation efficiency of MB under visible light. They achieved a degradation efficiency of 82% after 120 min. In addition, a silver surface-modified zinc oxide/cellulose acetate/polypyrrole (ZnO/CA/Ppy) nanocatalyst was developed for the degradation of MG. They achieved a degradation efficiency of 93.5% after 60 min. It can be seen from these examples that the photocatalyst has a significant effect on the degradation of the pollutant. On the other hand, the synthesis of high-efficiency photocatalyst heterostructures remains a great challenge for scientists.^27,28^

In this context, few studies have reported the synthesis of a SnO_2_/Zn_2_–SnO_4_ heterostructure by the sol–gel process. Moreover, the photocatalytic activity of the SnO_2_/Zn_2_–SnO_4_ photocatalyst has not been previously reported. In this study, SnO_2_/Zn_2_–SnO_4_ heterostructures were synthesized by the sol–gel process and tested for the degradation of MG dye by photocatalysis under ultraviolet light.

## 2 Materials and Methods

### 2.1 Chemicals

The following reagents from Sigma-Aldrich were used in the sol–gel synthesis: Tin­(IV) chloride pentahydrate (SnCl_4_. 5H_2_O, 99.8%), poly­(vinyl alcohol) (PVA, 99%), absolute ethanol (ETOH, 98%), zinc nitrate hexahydrate (Zn­(NO_3_)_2_·6H_2_O, 99%), ascorbic acid (AA, C_6_H_8_O_6_, 99%), sodium ethylene diamine tetraacetate (EDTA-2Na, C_10_H_16_N_2_O_8_, 99%), and isopropyl alcohol (IPA, C_3_H_8_O, 99.5%). Deionized water was also used in the syntheses.

### 2.2 Synthesis of SnO_2_/Zn_2_–SnO_4_ Heterostructure

First, 10 g of tin­(IV) chloride pentahydrate (SnCl_4_·5H_2_O) was dissolved in 50 mL of distilled water under continuous stirring to obtain a transparent solution. Then, poly­(vinyl alcohol) (PVA), ethanol (ETOH), and H_2_O were added and stirred for 30 min. These act as stabilizing agents and were gradually incorporated into the solution. The mixture was gradually heated up to 80 °C for 4 h to form a gel. It was then dried at 80 °C for 12 h and subsequently sintered for 3 h at a heating rate of 5 °C/min up to 900 °C. This process yielded plain SnO_2_.

Next, new (10 g of tin­(IV)) SnCl_4_. 5H_2_O solutions were prepared and mixed with zinc nitrate hexahydrate (Zn­(NO_3_)_2_·6H_2_O), varying the molar ratios, i.e., 1:2, 1:4, and 1:6. These were slowly added to the previous solution under continuous stirring for 30 min to ensure homogeneous dispersion. The mixture was also heated at 80 °C for 4 h to form a modified gel. It was then dried at 80 °C for 12 h and sintered for 3 h at a heating rate of 5 °C/min up to 900 °C to remove organic content and form powders. The synthesized materials were labeled as SnZn 1:2, SnZn 1:4, and SnZn 1:6,^29^ and the main synthesis steps are illustrated in two routes in Figure 1. In the present sol–gel synthesis of the SnO_2_/Zn_2_SnO_4_ heterostructure, a mixed solvent system composed of distilled water and ethanol was employed. Distilled water acted as the primary solvent for dissolving the metal precursors, SnCl_4_·5H_2_O and Zn­(NO_3_)_2_·6H_2_O, while ethanol was added as a cosolvent to control hydrolysis and condensation reactions, improve homogeneity, and reduce premature precipitation.

**Figure 1:**
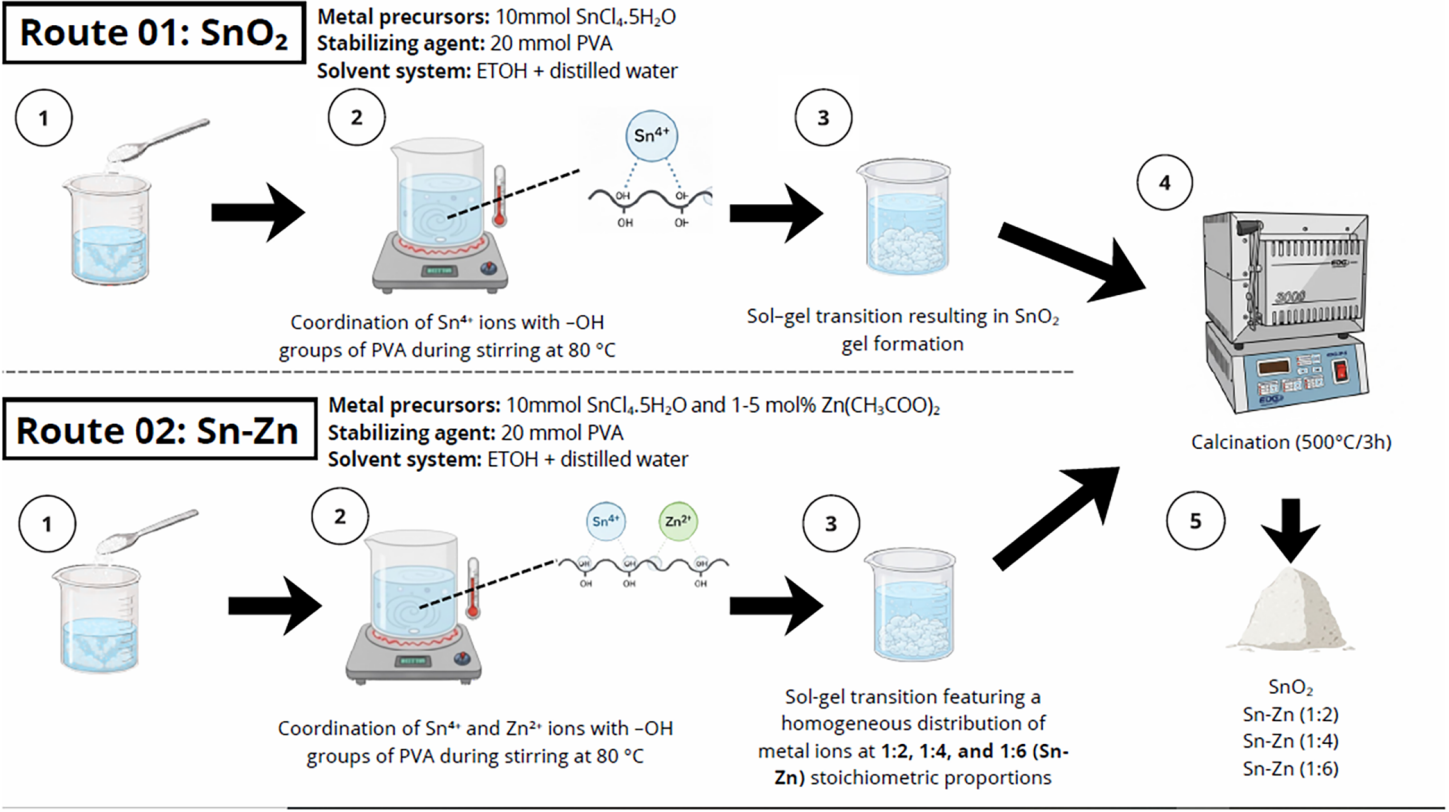
Schematic diagram of the synthesis process (by the author using Canva).

### 2.3 Physicochemical Characterization

The resulting powders were characterized by various methods. X-ray diffraction (XRD) was employed using an XRD-7000 Shimadzu diffractometer with Cu Kα radiation (1.5404 Å). The angular range (2θ) from 10° to 80° was scanned at a step of 0.02° s^–1^. Field emission scanning electron microscopy (FEG-SEM) coupled with energy-dispersive X-ray spectroscopy (EDS) was used to examine the particle morphology and the chemical composition of the materials. An SEM-FEG Zeiss Supra 35 VP equipped with a Bruker XFlash EDX was employed. N_2_ adsorption/desorption using the Brunauer–Emmett–Teller (BET) method was carried out using a BET Japan (Belsorp II mini) instrument, which was employed to evaluate the specific surface area of the materials. The thermal stability of the materials was evaluated by thermogravimetric analysis (TG) coupled with differential thermal analysis (DTA) using a DTG-60H instrument with simultaneous DTA-TG analyses. Visible diffuse reflectance spectroscopy using a UV–vis spectrophotometer (Shimadzu, UV-2600) between 200 and 900 nm was used to determine the optical properties of both pure and SnO_2_/Zn_2_–SnO_4_ materials. The charge transfer resistance of the materials was determined using electrochemical impedance spectroscopy (EIS) with a CS310 M potentiostat/galvanostat.

### 2.4 Photocatalytic Tests

The photocatalytic evaluation of the materials was performed by malachite green (MG) dye degradation. For this purpose, 100 mg of each sample was added to beakers containing 100 mL of a 10 ppm of MG solution. The beakers were then placed inside a photoreactor equipped with six UV lamps (Philips TUV, 15 W). Before light irradiation, the beakers were stirred in the dark for 30 min to reach adsorption–desorption equilibrium. During UV exposure, 3 mL aliquots of the solution were removed every 20 min, and absorbance was measured using a UV–vis spectrophotometer. The degradation efficiency was calculated using eq 1:

1 $$\eta \left(\%\right)=\left(\frac{C_{0}-C_{t}}{C_{0}}\right)\times 100$$

where *C*_0_ is the initial concentration and *C*_*t*_ is the concentration at time “*t.*” Furthermore, in order to observe the degradation rate, the degradation rate constant (*k*) was calculated by fitting the results to a pseudo-first-order linear model according to eq 2:

2 $$\ln\frac{C}{C_{0}}=k\times t$$

where *C* and *C*_0_ are, respectively, the concentration of the contaminant and the initial concentration of the dye before irradiation. Finally, the photocatalytic tests were performed in triplicate to increase the reliability of the results.

For the sample with the best photocatalytic performance, five reuse cycles were performed. Between cycles, washing with distilled water and drying in an oven at 100 °C were performed to recover the photocatalyst. After the last cycle, XRD analysis was performed to observe possible structural changes. The influence of superoxide (^•^O_2_^–^), holes (h^+^), and hydroxyl (^•^OH) radicals on the photocatalytic process was determined using reagents that act as inhibitors of these radicals. Ascorbic acid was used for ^•^O_2_^–^, sodium ethylene diamine tetraacetate (EDTA-2Na) was used for h^+^, and isopropyl alcohol was used for ^•^OH. The inhibition tests were performed under conditions similar to those of the photocatalytic tests, except for the addition of reagents to the solution.

The photocatalysis experiments were carried out using the photocatalyst dosage (0.5–1.5 g. L^–1^), the concentration of the solution (5–15 ppm), and the pH of the solution (2–10) in order to study the influence of these parameters on the degradation of malachite green. Additionally, a ZetaPlus ζ-potential analyzer (Brookhaven Instruments) was used to determine the zero-charge potential (pzc). The influence of the type of irradiation was observed under simulated visible light irradiation and sunlight. For the test under simulated visible light, the samples were placed under agitation in a degradation box containing six Philips brand lamps (TL-D 18*W*/54–765) at room temperature. For the sunlight test, samples were placed under agitation on an open terrace on January 19, 2026, between 9:00 and 11:00 AM, at coordinates *S* = 5°50′20″ and *W* = 35°12′3.5″. Finally, the results of one of the degradation tests performed in triplicate on the best sample were used for comparison with the results obtained in the inhibitor, dosage, pH, concentration, and irradiation type tests.

## 3 Results and Discussion

### 3.1 Structural Properties

The structural properties and phase identification of pure and the SnO_2_/Zn_2_–SnO_4_ heterostructure were performed by XRD (Figure 2). They were indexed to the ICSD card no. 39173, corresponding to the tetragonal structure of tin oxide with *p*42/*mm* space group, which is its most common and stable form.^29,30^ These results prove the successful synthesis of pure SnO_2_ nanoparticles by the sol–gel process. The XRD patterns of the zinc–tin oxide heterostructure (Figure 2b–d) were indexed to the ICSD card *n* ^o^ 28,235 and confirmed the presence of the cubic phase of Zn_2_SnO_4_ in all samples. The main diffraction peaks correspond to the crystallographic planes of the rutile-type tetragonal (cassiterite) structure of SnO_2_, indicating that the crystalline structure of SnO_2_ was preserved, even after the addition of Zn. Additional peaks are visible in the samples, which can be clearly attributed to Zn_2_SnO_4_ (as the cubic structure), suggesting that Zn^2+^ was substitutionally incorporated into the SnO_2_ lattice, replacing Sn^4+^ ions. Slight decreases in peak intensity can also be observed with increasing concentration of Zn (most evident between pure SnO_2_ and Zn_2_SnO_4_ material with 46.47% of Zn). This may indicate lattice deformation or compression caused by the difference in ionic radius between Sn^4+^ (∼0.71 Å) and Zn^2+^ (∼0.74 Å), consequently reducing the crystallite size or changes in lattice parameters, common in substitutional doping processes. It has been found that the addition of cations such as Mn^2+^, Fe^3+^, Cu^2+^, Co^2+^, Zn^2+^, among others, increases the specific surface area of SnO_2_, creating point defects that activate the densification of the powders.^23,31^ Rietveld refinement of the collected XRD data was performed for all samples in order to statistically prove the findings. Rietveld refinement showed that the obtained powders consisted of a mixture of Zn_2_SnO_4_ with a partially inverse spinel structure, space group *Fd*3*m* (71.4 wt %) and SnO_2_, space group *P*42/*mnm* (28.6 wt %), as shown. The Scherrer formula was employed to calculate the crystallite size (eq 3)­

3 $$D=\frac{k\lambda }{\beta \;\cos\theta }$$

where *k* is a constant with value 0.91; λ is the wavelength (*A*) of the incident X-ray beam; β is the full width at half-maximum (rad) and θ is the Bragg angle (°).

**Figure 2:**
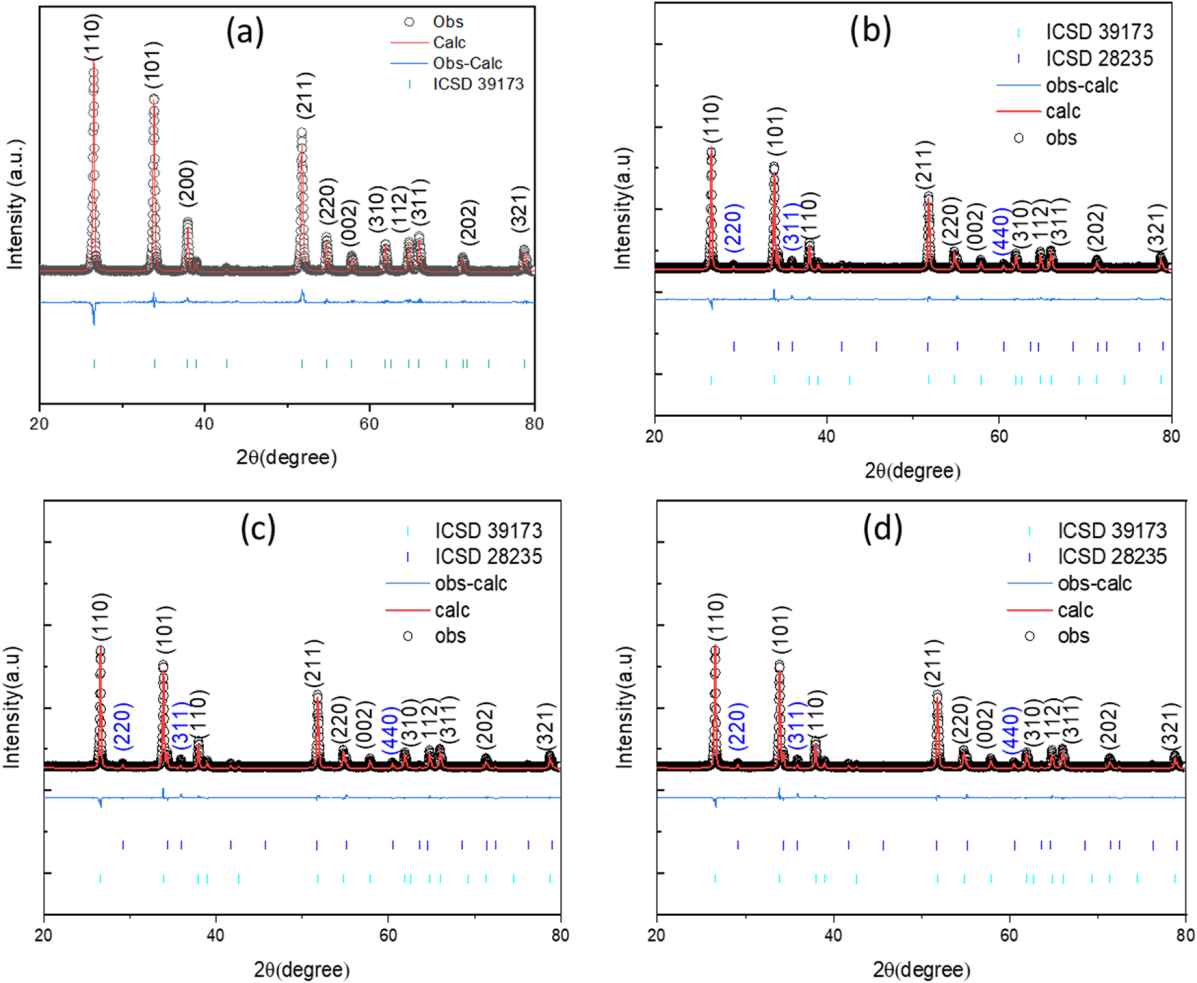
XRD patterns of (a) SnO_2_, (b) SnZn 1:2, (c) SnZn 1:4, and (d) SnZn 1:6.

Rietveld refinement of the XRD patterns shows that the peaks are asymmetric, and the quality of the refinements was significant based on the low values of χ^2^, *R*_wp_, and *R*_exp_ presented in Table 1. It was also observed that the introduction of Zn favored the change in crystallite size between the pure SnO_2_ structures.

**Table 1:** Rietveld Refinement Data of the Synthesized Samples

			lattice parameters (*A*)				quality of fit		
samples	phase	phase quantity (%)	*a*	*b*	*c*	crystallite size (nm	χ^2^	*R*_wp_	*R*_exp_
SnO_2_	SnO_2_	100.00	4.73	4.73	3.18	97.6174	1.36	14.03	10.28
ZnSn 1:2	SnO_2_	89.53	4.73	4.73	3.18	29.1618	1.53	14.9	9.87
	Zn_2_SnO_4_	10.46	8.65	8.65	8.65	13.9805			
ZnSn 1:4	SnO_2_	86.38	4.73	4.73	4.73	25.19427	1.52	14.9	9.89
	Zn_2_SnO_4_	13.61	8.65	8.65	8.65	10.28872			
ZnSn 1:6	SnO_2_	84.49	4.73	4.73	3.18	24.3130	1.43	13.9	9.6
	Zn_2_SnO_4_	15.50	8.65	8.65	8.65	14.1899			

### 3.2 Morphological Analysis

The morphology of pure SnO_2_ and Zn-SnO_2_ catalysts has been observed from the FE-SEM images presented in Figure 3. Figure 3a presents the SEM image of pure SnO_2_ nanoparticles as a mixture of spherical and tetragonal particles. The SEM image of the sample ZnSn 1:2 (Figure 3b) reveals a reduction of the particle size along with particle agglomeration. Figure 3c presents the EDS spectrum of the ZnSn 1:2 sample. This figure shows the characteristic peaks of O (oxygen), Sn (tin), and Zn, corroborating the addition of Zn during synthesis. The low intensity of the peak related to Zn suggests that its concentration is lower than that of Sn. However, it is important to notice that the nominal Sn/Zn precursor ratio of 1:2 suggests a higher Zn content than that of the Sn content. This was not observed after the synthesis and could be explained by the fact that EDS analysis reflects only the local elemental composition and does not directly represent the precursor stoichiometry or the phase distribution within the heterostructure.^32^ In the SnZn 1:2 sample, Zn is mainly involved in the formation of the Zn_2_SnO_4_ secondary phase and at the SnO_2_/Zn_2_SnO_4_ heterointerfaces, rather than forming Zn-rich segregated regions detectable by EDS. The relatively low Zn atomic percentage (0.63 atom %) indicates that Zn is structurally incorporated into the heterostructure, contributing to interfacial bonding and electronic coupling between SnO_2_ and Zn_2_SnO_4_.^33,34^ The distribution of the elements presented in the EDS maps (Figure 3d) reveals good homogeneity. In summary, the addition of Zn influenced particle morphology and agglomeration, resulting in smaller, agglomerated, and well-defined particles and porosity, suggesting good surface area. These characteristics are important for catalysis applications. The results confirmed the successful synthesis of the zinc tin oxide by the sol–gel method, and are in accordance with previous investigations.^32–34^

**Figure 3:**
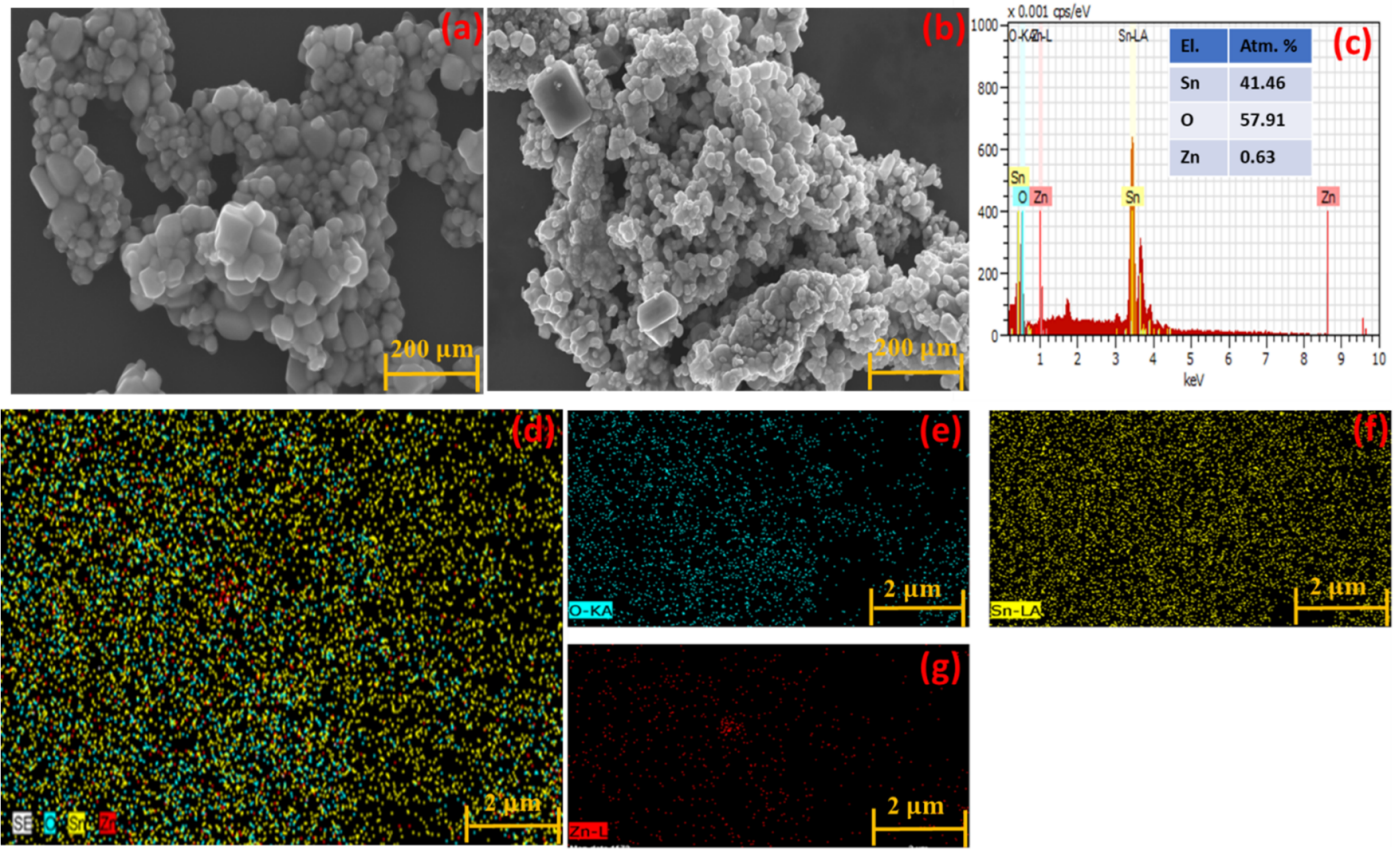
SEM images of (a) SnO_2_, (b) SnZn 1:2, (c) EDS graph, (d) elemental distribution, (e) oxygen, (f) tin, and (g) zinc.

### 3.3 Surface Area Measurements

The specific surface areas of the nanocatalysts were determined using the nitrogen (N_2_) adsorption–desorption technique based on the BET method. The results are presented in Figure 4, which shows similar behavior for all samples. The quantity of N_2_ adsorbed increases quickly at low relative pressures, followed by a steady step up to a relative pressure of 0.8 and finally a rapid increase. These results follow the adsorption isotherm type IV, and the model suggests that the adsorption of gas molecules increases with the relative pressure and presents an H3 hysteresis loop.^38^ The samples responsible for the adsorption could present two textures, notably microporous and mesoporous structures. As can be observed from the results (Table 2), all of the nanocatalysts exhibited a specific surface area with a slight difference. The total pore volume and average pore diameter of the samples are reported in Table 2. It is observed that the pore radius of the catalysts was in the range of 2–50 nm, which is the range of mesoporous materials.^38^ The as-prepared photocatalysts exhibited good specific surface area and pore radius, which could provide more available active sites and therefore would be beneficial for the photocatalytic process. The as-prepared photocatalysts exhibited good specific surface area and pore radius, which could provide more available active sites and therefore be beneficial for the photocatalytic process.^35,36^

**Figure 4:**
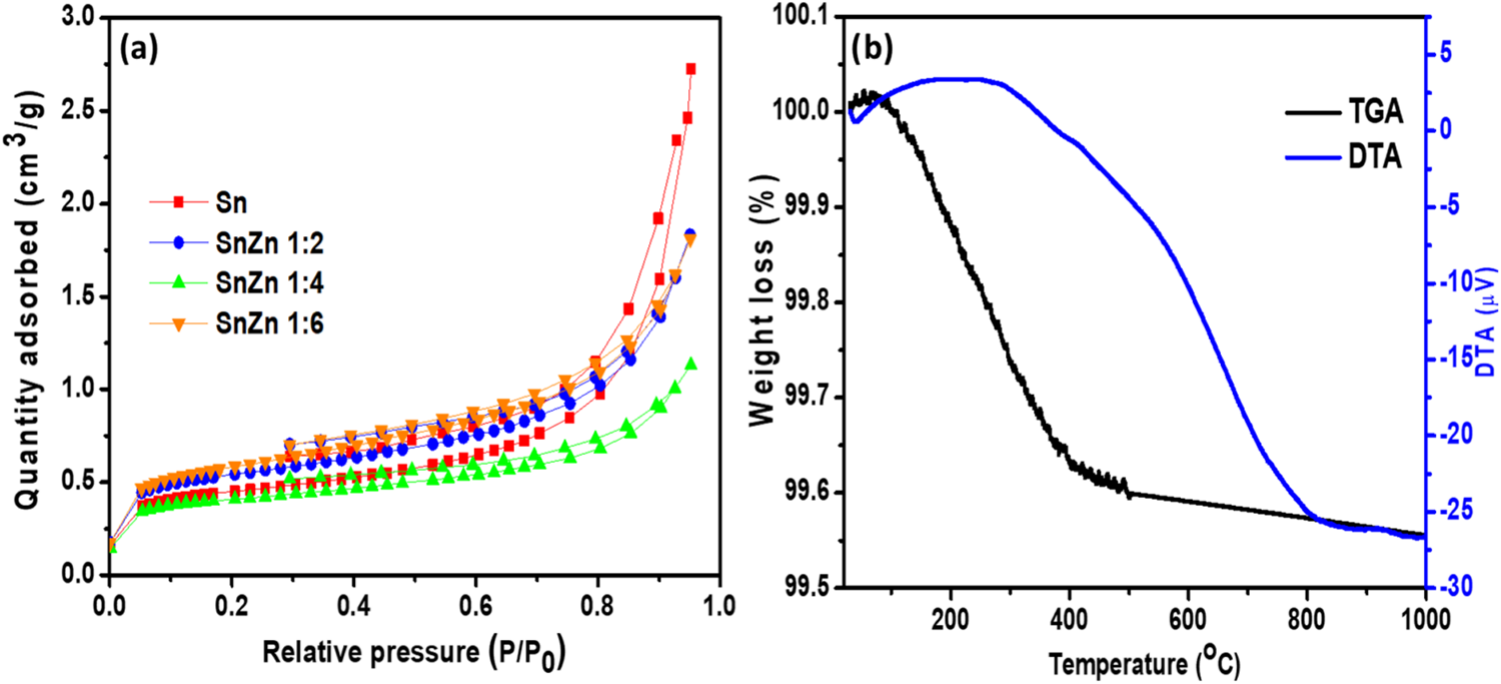
(a) Nitrogen adsorption/desorption curves of Sn, SnZn 1:2, SnZn 1:4, and SnZn 1:6 samples. (b) TGA-DTA curves of the SnZn 1:2 catalyst.

**Table 2:** BET Specific Surface Area of the Samples

samples	BET specific surface area (m^2^/g)	total volume (10^–3^ cm^3^/g)	average pore diameter (nm)
SnO	1.65	4.22	10.21
SnZn 1:2	1.98	2.83	5.72
SnZn 1:4	1.50	1.75	4.66
SnZn 1:6	2.14	2.80	5.24

### 3.4 Thermal Analysis

The thermal stability of the photocatalysts was studied by TGA-DTA analyses, and the results are depicted in Figure 4b. A small drop (∼1–2%) is observed at the beginning of the temperature range of 25–150 °C, accompanied by a slight endothermic shoulder, corresponding to the evaporation of physically adsorbed water and part of the hydration water from the precursors (SnCl_4_·5H_2_O and Zn­(NO_3_)_2_·6H_2_O). This does not affect the structure of the SnZn 1:2 material but indicates that the gel retains a considerable amount of water. From 150 to 250 °C, dehydroxylation/gel reorganization was noticed. Within this range, the TG curve exhibits a moderate and steady mass loss, accompanied by a slight endothermic inflection in the DTA curve. This is associated with the removal of ^–^OH groups and the rearrangement of the amorphous inorganic network.^28^ Between 250 and 350 °C, the decomposition of nitrates and chlorides took place. The curves show a significant mass loss (the main event in the TG) and a strong exothermic peak in the DTA plot. This confirms the decomposition of Zn­(NO_3_)_2_, the release of NO*_x_* gases, and possible liberation of HCl from tin complexes, as well as the combustion of organics from the sol–gel process. In the doping process, the higher the Zn content, the more intense the mass loss becomes due to the greater amount of nitrate present. In the temperature range 350–500 °C, the stabilization of the TG curve and the broader exothermic peak (∼450 °C) in the DTA could be attributed to the crystallization of SnO_2_ (cassiterite phase). This indicates the transition from the amorphous/hydroxide matrix to crystalline SnO_2_, with part of the Zn being incorporated into the lattice or forming fine ZnO particles. Above 500 °C, the TG curve becomes practically stable, with a slight drop.^29^

### 3.5 Optical and Electrochemical Analysis of the Photocatalyst

The energy band gap of the nanocatalysts was determined from the UV–vis diffuse reflectance spectrum and Tauc plot (Figure 5a,b, respectively). The energy band gap (eq 4) is one of the important parameters to address the photocatalytic ability of a material, from the absorbance obtained in UV–vis tests.^37^

4 $${\left(\alpha \mathrm{hv}\right)}^{m}=B\left(E_{f}-E_{g}\right)$$

where *m* = 1/2 or 2 for the direct and indirect allowed, respectively, *B* = constant, *h* = Planck’s constant, *E*_f_ = photon energy, and *E*_g_ = energy band gap.^39^

**Figure 5:**
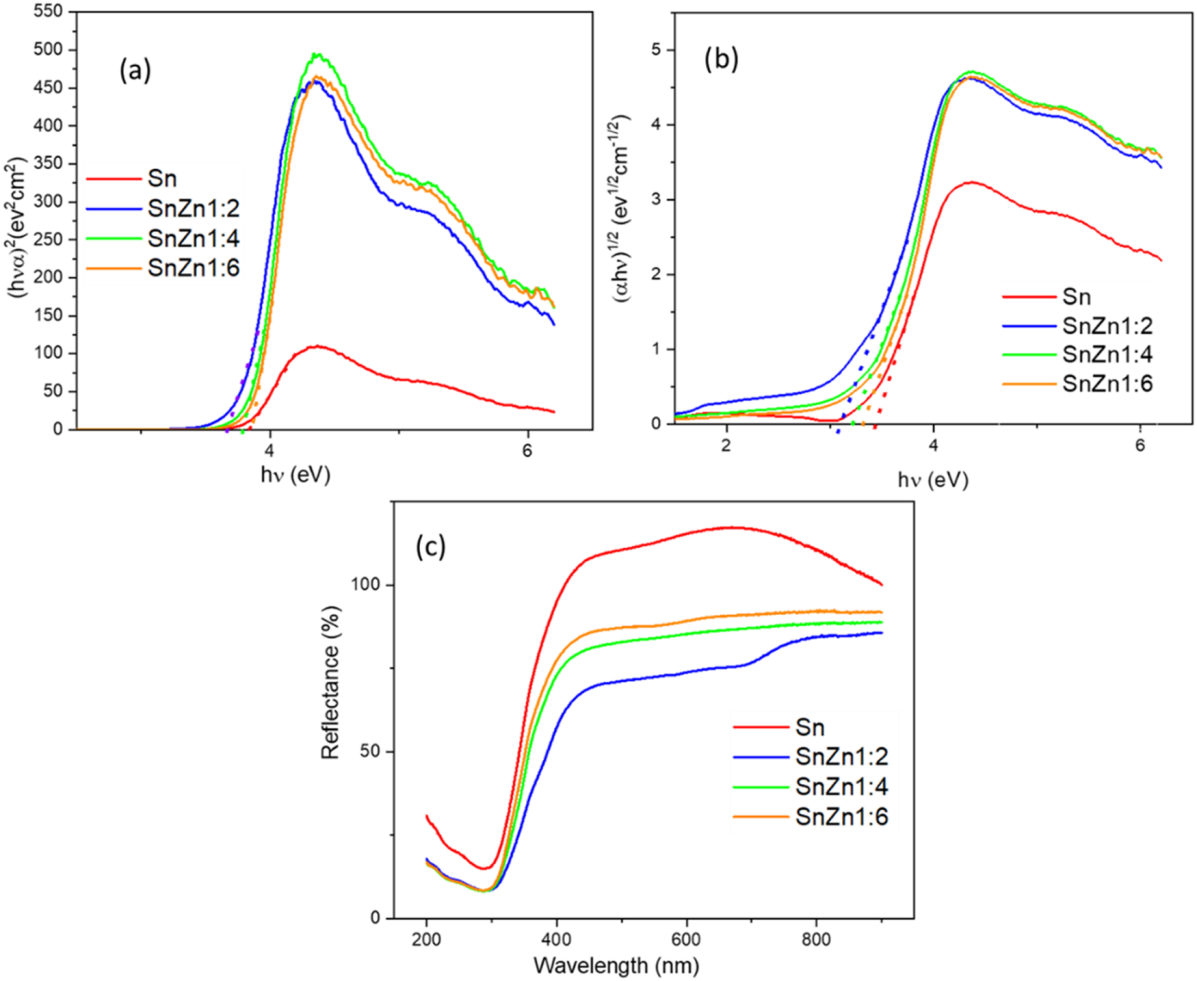
(a) UV–vis and (b, c) diffuse reflectance spectra band gap calculation of the Sn, SnZn 1:2, SnZn 1:4, and SnZn 1:6 nanocatalysts.

Table 3 shows the values of the direct and indirect allowed energy band gaps obtained from eq 4. It can be seen that the resulting energy band gap decreased with the addition of Zn.

**Table 3:** Energy Band Gap of the Samples

catalysts	wavelength (nm)	α^1/2^ direct (eV)	α^2^ indirect (eV)
Sn	296.78	3.33	4.45
SnZn 1:2	297.80	2.88	3.57
SnZn 1:4	304.07	3.27	3.64
SnZn 1:6	302.32	3.30	3.73

The increase in electrical conductivity is mainly associated with the presence of intrinsic defects, such as oxygen vacancies, the modulation of the electronic density of states, and the formation of heterostructures, which contribute to an increased charge carrier concentration and improved electronic transport within the material.^40,41^ These defects act as shallow donor levels, facilitating electronic conduction and suppressing the recombination of photoinduced charge carriers.^38,39^ In this context, electrical conduction occurs predominantly by electronic transport, where photoexcited electrons are promoted from the valence band to the conduction band under light irradiation.^43^ Structural defects, including stacking faults, modify the local electronic structure and introduce additional electronic states within the band structure, thereby enhancing charge separation and interfacial charge transfer processes, as previously reported.^44^ Consequently, the reduction of the SnO_2_ band gap enhances visible light absorption and further suppresses electron–hole recombination, resulting in improved photocatalytic activity and higher degradation efficiency of organic dyes using the as-prepared photocatalysts.^42–44^

In Figure 5c, it is observed that the curve for Sn shows the highest reflectance in the longer wavelength regions compared with that of the heterostructure materials. The reflectance values of the SnZn 1:2, SnZn 1:4, and SnZn 1:6 materials are below that of pure Sn along the visible light range, indicating that doping improves light absorption. The increase in reflectance around ∼350–400 nm for all samples may indicate the absorption edge. The heterostructure exhibits a slightly smoother or shifted rise, which may suggest a change in the absorption edge (optical band gap) or increased scattering/defects causing residual absorption.^48^

The lower band gap energy observed for the Sn:Zn (1:2) sample compared with the SnO_2_:ZnSn (1:4) and Sn:Zn (1:6) samples can be attributed to the synergistic effects of optimal heterojunction formation, electronic structure modification, and defect-induced states. At the Sn:Zn (1:2) molar ratio, the intimate interfacial contact between the SnO_2_ and Zn_2_SnO_4_ phases promotes strong electronic coupling, facilitating effective band alignment and charge transfer across the heterostructure interface. This interaction leads to band bending and the formation of intermediate energy levels near the conduction band, resulting in an apparent narrowing of the optical band gap.^49^ Additionally, the lower Zn content in the 1:2 composition favors a higher density of oxygen vacancies and structural defects, which introduce localized states within the forbidden band. These defect states enhance visible light absorption and further reduce the effective band gap energy. In contrast, higher Zn contents (1:4 and 1:6) promote a more stoichiometric Zn_2_SnO_4_ phase with a reduced defect density and weaker interfacial interaction with SnO_2_. This leads to a partial recovery of the intrinsic wide band gap characteristic of both SnO_2_ and Zn_2_SnO_4_, resulting in higher band gap energies.^50^ Therefore, the reduced band gap energy of the Sn:Zn (1:2) sample arises from the combined effects of optimized heterostructure formation, enhanced interfacial charge transfer, and defect-mediated electronic states.^51^

In the context of photocatalysis, the electrochemical analysis of EIS is important because the Nyquist radius is related to the charge transfer rate at the photocatalyst interface. Thus, a smaller arc radius represents less resistance to charge transfer.^49,50^ The impedance spectra presented in Figure 6 show that all SnZn composites exhibited a radius smaller than that of SnO_2_. Knowing that the photocatalytic process is improved in materials with lower band gap energy, larger specific surface area, and higher charge transfer rate, it is expected that the SnZn samples, especially the 1:2 ratio sample, will exhibit better photocatalytic performance compared with pure SnO_2_.^45–47^

**Figure 6:**
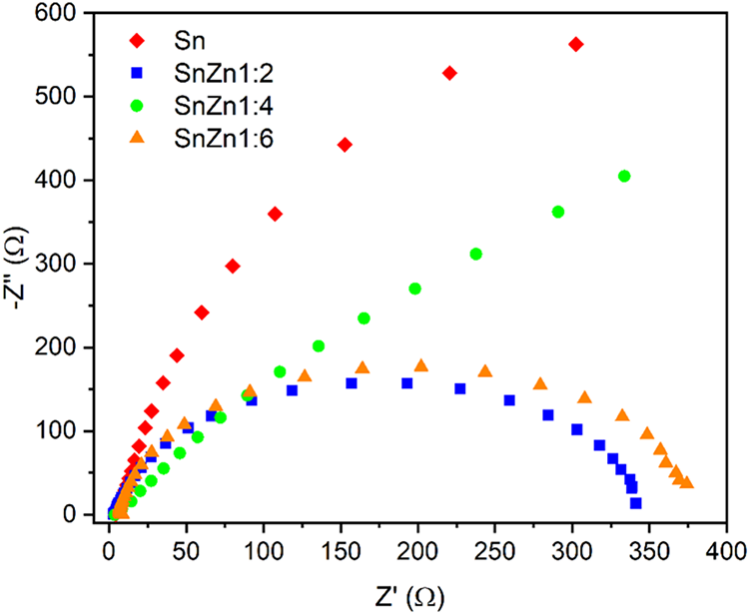
Electrochemical impedance spectra of Sn, SnZn 1:2, SnZn 1:4, and SnZn 1:6 nanocatalysts.

### 3.6 Photocatalytic Degradation of Malachite Green

The photocatalytic performances of the SnO_2_ and SnZn (SnO_2_/Zn_2_–SnO_4_) catalysts were evaluated by the degradation of the malachite green (MG) dye under ultraviolet light. As shown in Figure 7a, there was 25% photolysis, indicating a minimal influence of pollutant sensitization on the process. With the presence of photocatalysts, there was a considerable increase in efficiency, especially for the SnZn 1:2 sample, which showed over 96% MG removal after 100 min of UV light exposure. The relevant performance of this sample was confirmed by the degradation constant *k* (Figure 7b), which was superior to that of pure SnO_2_ and the other samples. Five reuse cycles were then conducted under the same degradation test conditions. As shown in Figure 7c, there was only a 33% decrease in photocatalytic efficiency at the end of the last cycle. Furthermore, no structural changes were observed in the photocatalyst when comparing the XRD before and after the reuse cycles (Figure 7d), indicating that the material obtained presents good structural and photocatalytic stability with successive ultraviolet light exposure.^52,53^

**Figure 7:**
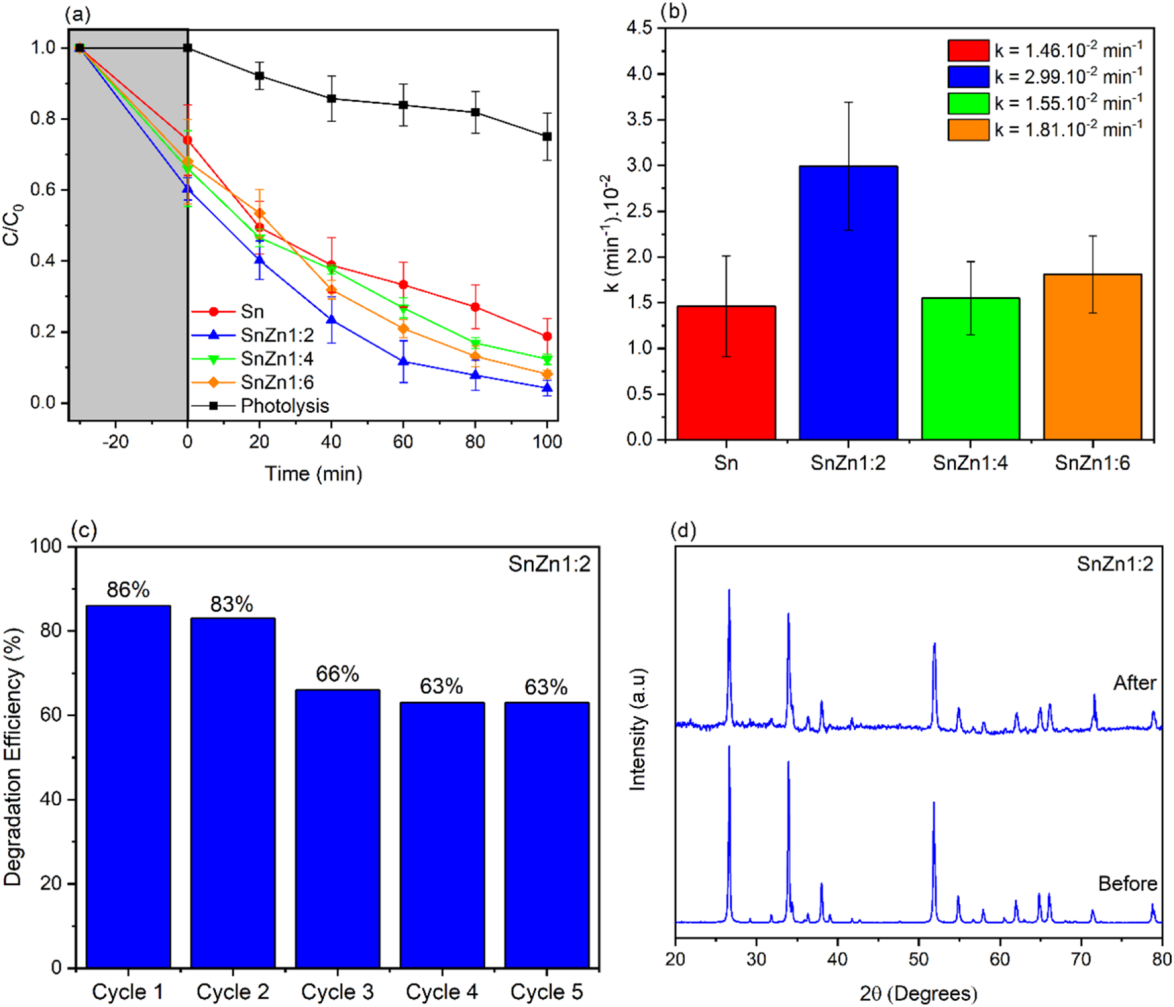
(a) Photocatalytic degradation of MG; (b) rate constants of photocatalytic degradation of MG; (c) results of reuse cycles of the SnZn 1:2 heterostructure; (d) diffraction patterns before and after five reuse cycles.

The influence of active radicals on degradation was investigated by photocatalytic tests using inhibitors in conjunction with the SnZn 1:2 sample. The results can be seen in Figure 8, which shows that except for the AA reagent, the addition of the inhibitor reagents reduced the photocatalytic efficiency, demonstrating that h^+^ and ^•^OH are important for the photocatalytic process.

**Figure 8:**
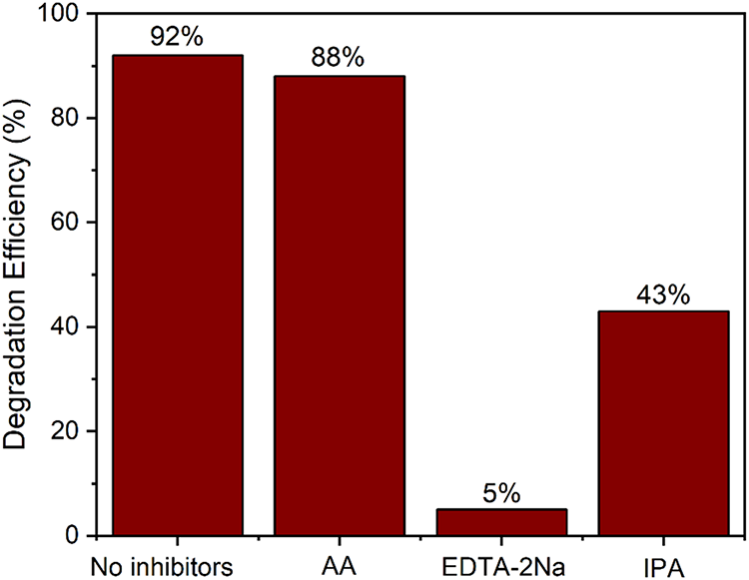
Inhibition test results.

From Table 4, it is possible to compare the photocatalytic activity results presented herein with those of similar SnO_2_-based materials. It can be observed that the addition of Zn is the one that best combines photocatalytic performance with light exposure time, which reveals the efficiency and quality of the photocatalyst produced. This performance could be explained by the structural properties of the materials, which exhibited two main phases: the low crystallite size obtained by the XRD analyses and the good specific surface area of the photocatalyst. These characteristics further contribute to enhancing the degradation efficiency by providing more active sites for the photocatalytic process.

**Table 4:** Photocatalytic Activity of SnO_2_/Zn_2_–SnO_4_ Heterostructures

photocatalyst	dye	degradation efficiency	time	refs
CdS/SnO_2_	MB^a^	90%	180 min	^54^
g-C_3_N_4_/rGO/SnO_2_	RhB^b^	83%	120 min	^55^
SnO_2_/CuO	MB^a^	90%	180 min	^56^
SnO_2_/TiO_2_	MB	93%	180 min	^26^
SnO_2_/ZnO	MV^c^	91%	120 min	^57^
SnO_2_/ZnO	MB	82%	120 min	^58^
SnO_2_	MB	98.56%	30 min	^59^
SnO_2_	MB	97.84%	50 min	^59^
SnO_2_	MB	97.12%	80 min	^59^
SnO_2_-NPs	MO	98.26%	40 min	^59^
SnO_2_-NPs	MO	97.39	70 min	^59^
SnO_2_-NPs	MO	96.52	100 min	^59^
BFOT30	MB	97%	70 min	^60^
TDO	MB	99.35%	50 min	^61^
TDO	RhB	99.28%	60 min	^61^
ZIF-8/Ni, Co e Cu	RhB	20%	1440 min	^62^
SnO_2_/Zn_2_–SnO_4_	MG	96%	100 min	this work

a MB = Methylene blue; ZIF-8 = zeolitic imidazolate framework-8; MO = Methyl orange.

b RhB = Rhodamine B.

c MV = Methyl violet; TDO = Titanium dioxide; BFOT30 = BiFeO_3_/TiO_2_.

The study of parameters related to the concentration of the pollutant solution, the dosage of the photocatalyst, and the pH of the medium is of great relevance, since they allow the determination of the ideal quantities to maximize degradation efficiency, in addition to providing information on the protonation or deprotonation state of the pollutants in solution.^56,57^ Figure 9 shows the results of the effect of variations in these three parameters on the photocatalytic performance. As shown in Figure 9a, in the dosage test, there was a progressive increase in degradation efficiency, thanks to the increase in the number of active sites in the photocatalyst and, consequently, the number of redox radicals.^65^ In the concentration test (Figure 9b), the degradation efficiency decreased with the increase in the concentration of malachite green, due to the limited number of active sites on the surface of the photocatalyst in relation to the increase in pollutant molecules.^66^

**Figure 9:**
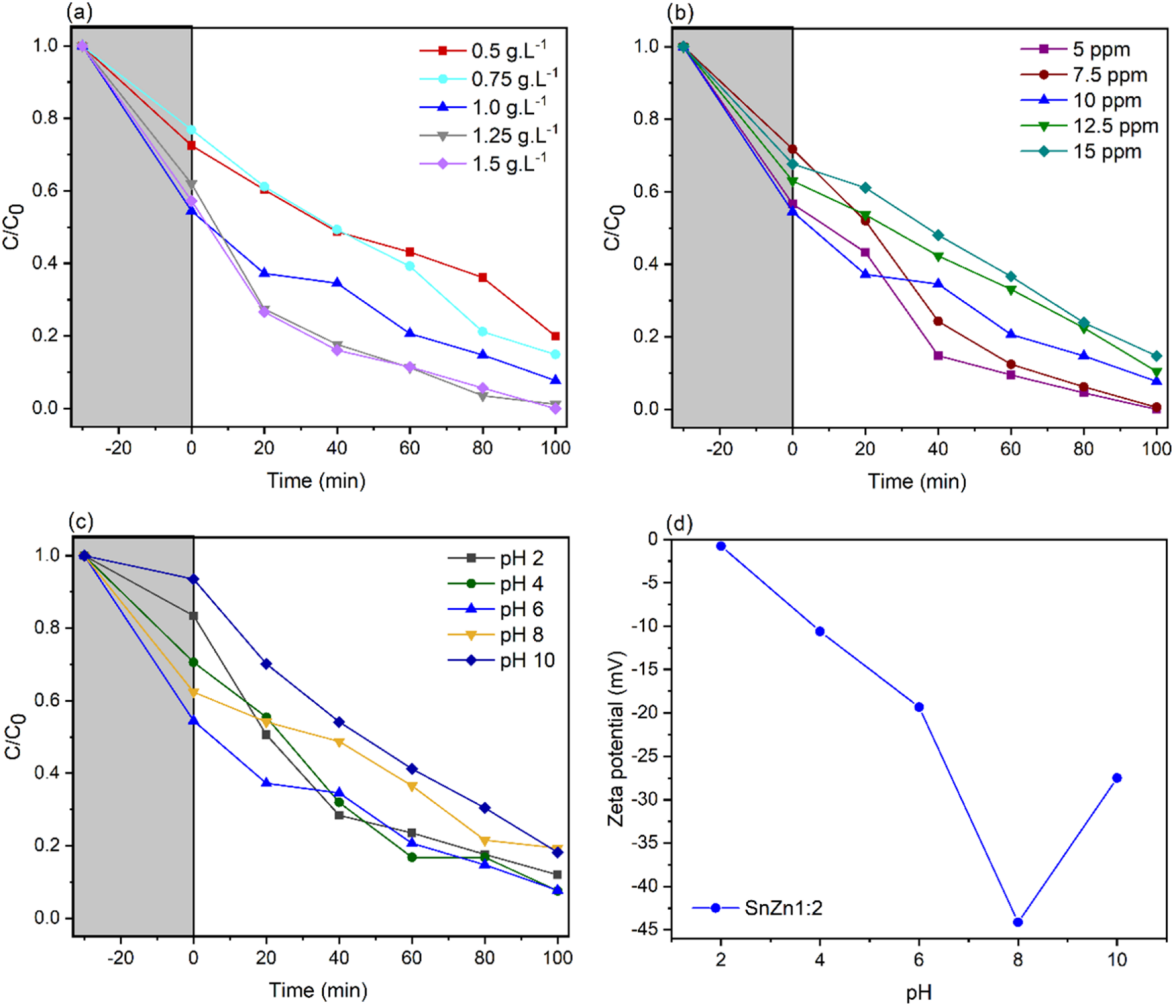
Photocatalytic tests of the dosage (a), concentration (b), and pH (c) of the malachite green dye. ζ-Potential (d) of SnZn 1:2.

The results of the pH variation tests shown in Figure 9c reveal that the best performance occurred at pH 6 (natural pH of the solution) and pH 4. This can be explained based on the dissociation constants (p*K*_a_) of the contaminant and the potential for zero charge (pzc) of the SnZn 1:2 sample. Malachite green has a p*K*_a_ of 6.9.^67^ This means that at pH < p*K*_a_, the dye will be in its protonated form with a positive charge, while at pH > p*K*_a_, the dye will be deprotonated and negatively charged.^68^ Regarding pzc, Figure 9d shows that SnZn 1:2 has a negative charge at all measured pH values. Therefore, since MG has positive charges at pHs 4 and 6, the degradation results are justified, as there is no repulsion between the photocatalyst and the dye. Finally, the low efficiency shown at pH 2, even with the protonated dye, is possibly due to the presence of excess H^+^ ions, which made the pollutant molecules more stable and resistant to degradation.^69^

In order to observe the photocatalyst’s performance under other light sources, tests were carried out under simulated visible light irradiation and sunlight. The results in Figure 10 show that under visible light irradiation, the efficiency was reduced to 75%, while under sunlight exposure, 100% of the dye was removed after 80 min. The decrease in photocatalytic efficiency under visible light occurred because of the lower amount of UV light available in the lamp composition, which is less than 2% (approximately 2.16 W). According to Neppolian et al.,^69^ under similar irradiation powers, UV irradiation is more effective than visible irradiation because UV energy is higher than the band gap of photocatalysts, favoring the excitation of electron/hole pairs. Regarding the test under sunlight irradiation, Ollis et al.^70^ state that the higher the light intensity (irradiation power), the more photons there will be per unit time and unit area. Since the power of sunlight during the test period varied between 1000 and 1100 W·m^–2^, the activation of photons on the surface of the photocatalyst was maximized, as was the photocatalytic capacity, thus justifying the result obtained.^63,64^

**Figure 10:**
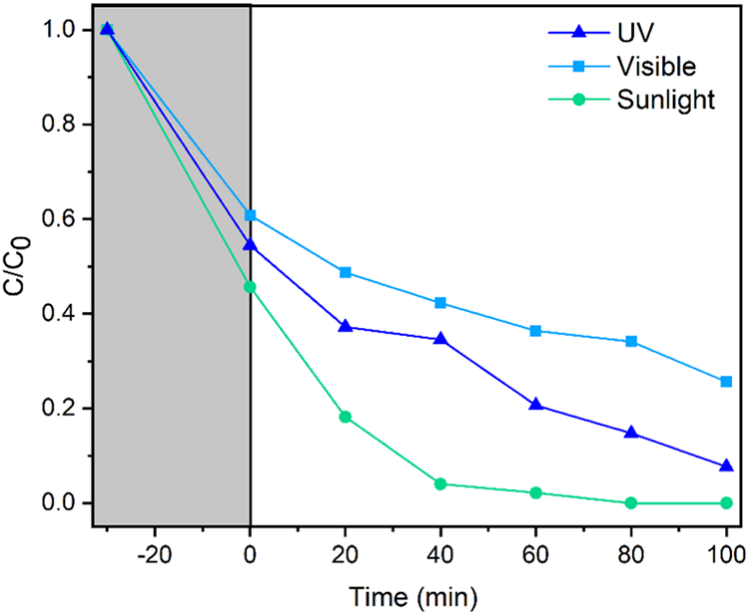
Results of MG degradation under ultraviolet light irradiation, simulated visible light, and sunlight.

To define the photocatalytic mechanism of SnO_2_/Zn_2_–SnO_4_ materials presented in Figure 11, the energies of the conduction band (CB) and valence band (VB) of SnZn 1:2 must initially be calculated, using eqs 5 and 6:^71^

5 $$\mathrm{CB}=\chi -E_{0}-0.5E_{g}$$

6 $$\mathrm{VB}=\mathrm{CB}+E_{g}$$

where *E*_0_ is the energy of free electrons on the hydrogen scale (4.5 eV), *E*_g_ is the band gap energy, and χ is the electronegativity of the semiconductors, which is calculated by eq 7:^72^

7 $$\chi ={\left[\left({x_{1}}^{n_{1}}\right)\times \left({x_{2}}^{n_{2}}\right)\right]}^{1/n_{1}+n_{2}}$$

where *n* is the number of atoms of element (*x*) in the compound. Therefore, for SnZn 1:2, the values of χ, CB, and VB are 6.1, 0.02, and 3.17 eV, respectively.

**Figure 11:**
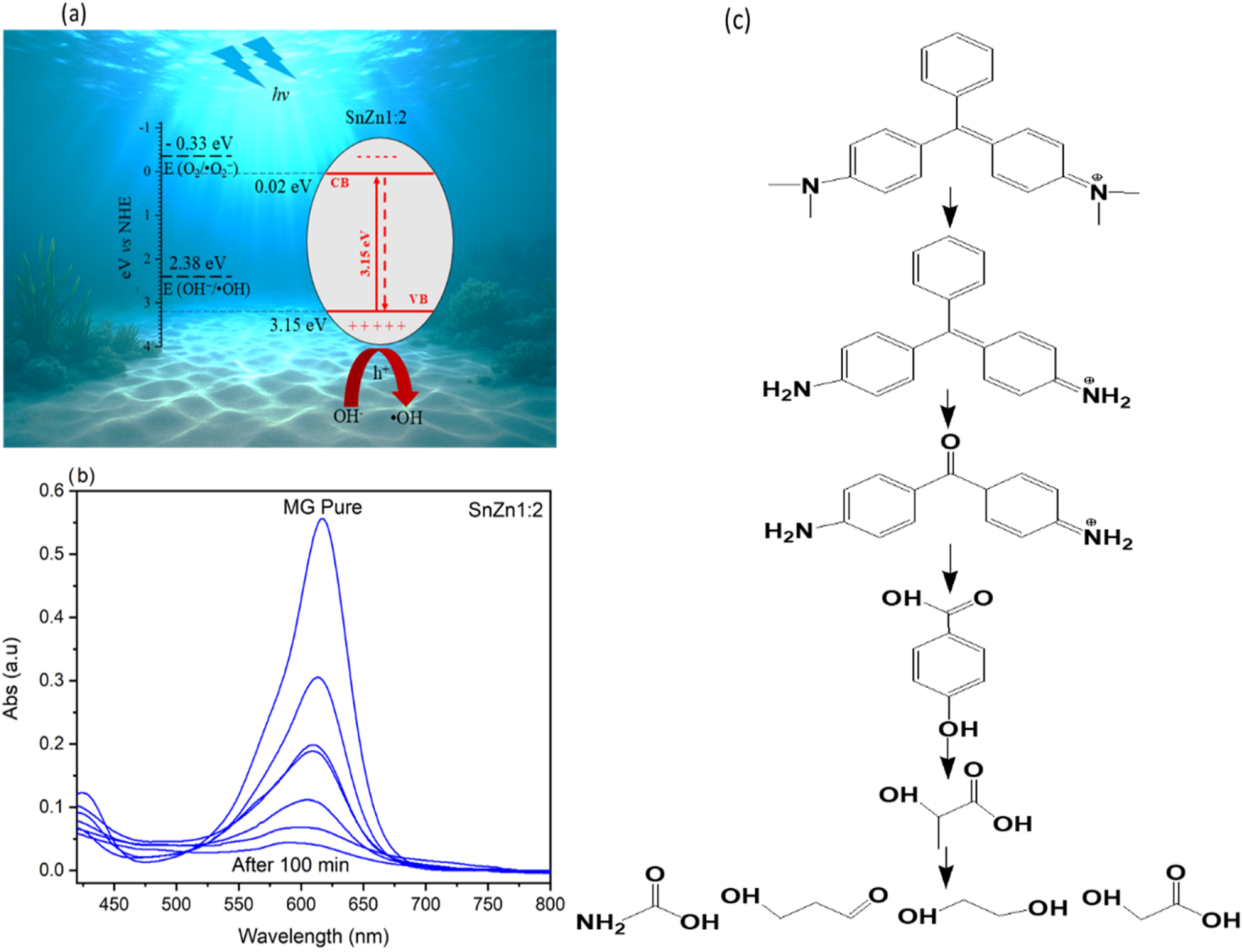
(a) Proposed mechanism for the photocatalytic activity of the SnZn 1:2 sample, (b) visible absorption spectra of malachite green versus reaction time, and (c) plausible mechanism of photocatalytic degradation of malachite green using SnZn 1:2 material.

With the values of CB, VB, and *E*_0_, an illustrative scheme of the photocatalytic mechanism of the SnZn 1:2 sample is shown in Figure 11a. It is observed that the conduction band (0.02 eV) is less negative than the O_2_/^•^O_2_^–^ reduction potential (−0.33 eV),^73^ which means that the photogenerated electrons do not react with O_2_ to form superoxide radicals (^•^O_2_^–^). Furthermore, the valence band (3.17 eV) is more positive than the OH^–^/^•^OH oxidation potential (2.38 eV).^74^ This means that the photogenerated holes in SnO_2_ can oxidize OH^–^ and form ^•^OH radicals. The photogenerated holes can also react with adsorbed water molecules on the surface of the SnZn photocatalyst to generate ^•^OH radicals. These oxidizing species act as strong oxidants, which attack and degrade the malachite green. These conclusions are consistent with the results of the inhibition tests. Therefore, after UV light irradiation, the hydroxyls generated by the holes break down the molecules present in the dye solution due to oxidation reactions. Eqs 8–11 summarize the role of radicals in photocatalysis.

8 $${\mathrm{Sn}}_{\mathrm{Zn}}+\mathrm{hv}\to {\mathrm{Sn}}_{\mathrm{Zn}}\left(e^{-}\right)+{\mathrm{Sn}}_{\mathrm{Zn}}\left(h^{+}\right)$$

9 SnZn(h+)+OH−→OH•

10 SnZn(h+)+H2Oads→OH•+H+

11 OH•+MG→CO2+H2O+smallinorganicmolecules

UV–vis absorption spectra of malachite green at different reaction times were obtained using the SnZn 1:2 photocatalyst. The results are presented in Figure 11b. This study was conducted to investigate the transformation pathway of malachite green during the process. This figure shows the decrease in the absorption peak (618 nm), which disappears completely after 100 min of the process. Another adsorption peak was found at around 600 nm, which could be assigned to the product formed after the demethylation reaction as reported previously.^75^ In the visible range, a decrease in the intensity peaks at 400 nm is observed, indicating the destruction of the skeleton of malachite green during the oxidation process. It is reported that during the reaction time, the intermediates could be further oxidized into small compounds by the hydroxyl radicals following the hydroxylation reaction.^24,76^ Based on these observations and the scavenger results and literature,^76^ a possible mechanism of degradation of malachite green using the SnZn 1:2 catalyst has been proposed and presented in Figure 11c.

## 4 Conclusions

This work reports the synthesis and characterization of the SnO_2_/Zn_2_–SnO_4_ heterostructure for the photocatalytic degradation of malachite green in aqueous medium under ultraviolet irradiation. The structural properties of the nanocatalysts revealed the formation of tetragonal phases of SnO_2_ and cubic phase of Zn_2_SnO_4_. The homogeneous distribution of the elements was observed by SEM. BET results revealed that the SnO_2_/Zn_2_–SnO_4_ catalyst has a mesoporous structure, with slight porosity, which was observed from the SEM analyses. The nature of defects, modulation of the density of electronic states, and presence of heterostructures enhance electrical conductivity. The photocatalysts exhibited a mesoporous structure with an average pore radius in the range of 2–50 nm. The band gap energy decreased and the charge transfer rate improved with increasing zinc concentration. These two characteristics are important for applications in photocatalysis. The photocatalysis degradation tests of malachite green indicated that SnZn 1:2 exhibited the highest degradation efficiency (96%) after 100 min of UV exposure. This result was attributed to the low band gap energy of the sample, which promotes easy electron movement during the photocatalytic process. The SnO_2_/Zn_2_–SnO_4_ photocatalyst indicated promising results and good stability in the degradation of dye and, therefore, could be employed in the treatment of water contaminated by organic pollutants in order to address environmental concerns.
